# Incidence and predictors of reoccurrence of opportunistic infection among adult HIV/AIDS patients attending ART clinic at public health facilities in Arba Minch town, southern Ethiopia: A retrospective cohort study

**DOI:** 10.1371/journal.pone.0261454

**Published:** 2021-12-31

**Authors:** Maycas Dembelu, Mesfin Kote, Girma Gilano, Temesgen Mohammed

**Affiliations:** 1 Department of Nursing, Faculty of Health Science, Mettu University, Mettu, Ethiopia; 2 School of Public Health, College of Medicine and Health Science, Arba Minch University, Arba Minch, Ethiopia; 3 Department of Public Health, Arba Minch College of Health Science, Arba Minch, Ethiopia; Tulane National Primate Research Center, UNITED STATES

## Abstract

**Background:**

Human immunodeficiency virus (HIV) infected individuals are prone to opportunistic infections (OIs) due to HIV mediated immune suppression. When opportunistic infections occur in the form of relapse or reinfection, it is said to be reoccurrence. This study was aimed to assess Incidence and predictors of reoccurrence of opportunistic infections among adult people living with HIV (PLHIV) attending ART clinics in Arba Minch Town, Southern Ethiopia

**Methods:**

This retrospective cohort study was conducted on 450 HIV/AIDS patients attending anti-retro viral therapy (ART) clinics in Arba Minch town, southern Ethiopia. Simple random sampling technique was used. Kaplan-Meier graph and log rank test were used for group wise comparison. Bivariate and multivariable Cox Proportional Hazard Regression model were used to identify independent predictors of reoccurrence of opportunistic infection.

**Result:**

One hundred nineteen HIV/AIDS patient had reoccurrence of opportunistic infection. The incidence rate was 11.5 per 1000 person months. The mean time of reoccurrence was 56 months. One of the most reoccurred OIs was pulmonary tuberculosis (PTB). Predictors that were associated significantly were recent cell differentiation 4 (CD4) count, recent body mass index (BMI), recent functional status, and duration on anti-retroviral therapy (ART).

**Conclusion:**

Though the incidence rate of OIs decreased from previous findings, attention should be given to HIV patients with low CD4 count, low BMI and for those bedridden patients.

## Introduction

Having claimed more than 32 million lives so far, Human immunodeficiency virus (HIV) continues to be a major global public health issue. There were approximately 37.9 million people living with HIV (PLHIV) at the end of 2018 [1, 2]. Of all PLHIV, Over two third (25.7 million) live in the Africa region [3]. Globally, one in three people living with HIV present to care with illness like recurrent opportunistic infection. According to World Health Organization (WHO) report, 300,000 people died due to acquired immunodeficiency syndrome (AIDS) related illness in Eastern and South Africa region [1, 3].

Human immunodeficiency virus positive individuals are prone to opportunistic infections (OIs) due to HIV mediated immune suppression [4]. When these opportunistic infections occur in the form of either reinfection or relapse, it is said to be reoccurrence [5]. The reoccurrence of pulmonary tuberculosis (PTB) infection is one of the morbidities which affect HIV patients the most. The rate of TB infection in HIV/AIDS patient is much higher than that of HIV uninfected individuals [6]. Again, the rate of reoccurrence is higher in HIV positive individuals. Furthermore, this reoccurrence characterized by the development of drug resistant mycobacterium tuberculosis, which makes caring for HIV patient with TB more difficult. This will increase the burden of treating HIV/TB co infected patient [7, 8].

The same is true for other opportunistic upper respiratory tract infectious diseases. For instance, HIV patients who are infected with recurrent upper respiratory bacterial and viral infections, even if the infection can be cured spontaneously or with treatment, no protective immunity is built. Thus, patients that are cured at the end of therapy can be infected with a new strain, and might be still at risk to develop severe and complicated diseases [9]. Likewise, recurrent opportunistic infections (OIs) of central nervous system (CNS) will be very difficult to manage with high morbidity and mortality in acute phase of the disease [10].

In Ethiopia, according to a hospital based study conducted in Debre Markos, the overall recurrence rate of OI was 75%, and the most common OIs were recurrent upper respiratory tract infection, chronic diarrhea, and pneumonia. Poor health care facility with regard to OIs treatment, poor adherence to anti-retroviral therapy (ART), inaccessible OI prophylaxis, presence of anemia, clinical stage of the disease, and low cell differentiation 4 (CD4) count makes the problem worse. These factors were also significant predictors for reoccurrence of OIs in this facility based study [11].

As far as our knowledge is concerned, little is known about the reoccurrence of opportunistic infections among HIV/AIDS patients attending ART in the study area. This study incorporates variables like duration on ART and regimen change, which previous studies did not address.

## Methods and materials

### Study design

A facility based retrospective cohort study design was applied by reviewing medical records of HIV/AIDS patients on ART.

### Study setting

Arba Minch town is located in Gamo zone, South Western Ethiopia. Arba Minch town has two sub cities, namely Secha and Sikela. The town has four public health facilities; Arba Minch General Hospital, Secha Health Center, Arba Minch Health Center, and Weze Health Center. The first three of these public health facilities provide ART service to HIV/AIDS patients. These public health facilities treat HIV patients with “test and treat” treatment protocol in their ART center as per national treatment guideline, which was introduced three years ago at national level. These three public health facilities, which provides ART service, were purposively included into the study. The study was conducted from 5 April, 2020 to15 August, 2020.

### Study population

All adult HIV/AIDS patient on ART follow up from September 11, 2013 to September 15, 2018 and who were previously diagnosed for opportunistic infection.

### Sample size, sampling procedure, and eligibility criteria

Based on the finding from the previous study, the sample size for current study was calculated. Magnitude of reoccurrence of OI was 75% [11] with 1.96 coefficient of reliability and 4% margin of error. By using single population formula and by considering 10% contingency the final sample size for this study was 494.

**Fig 1:**
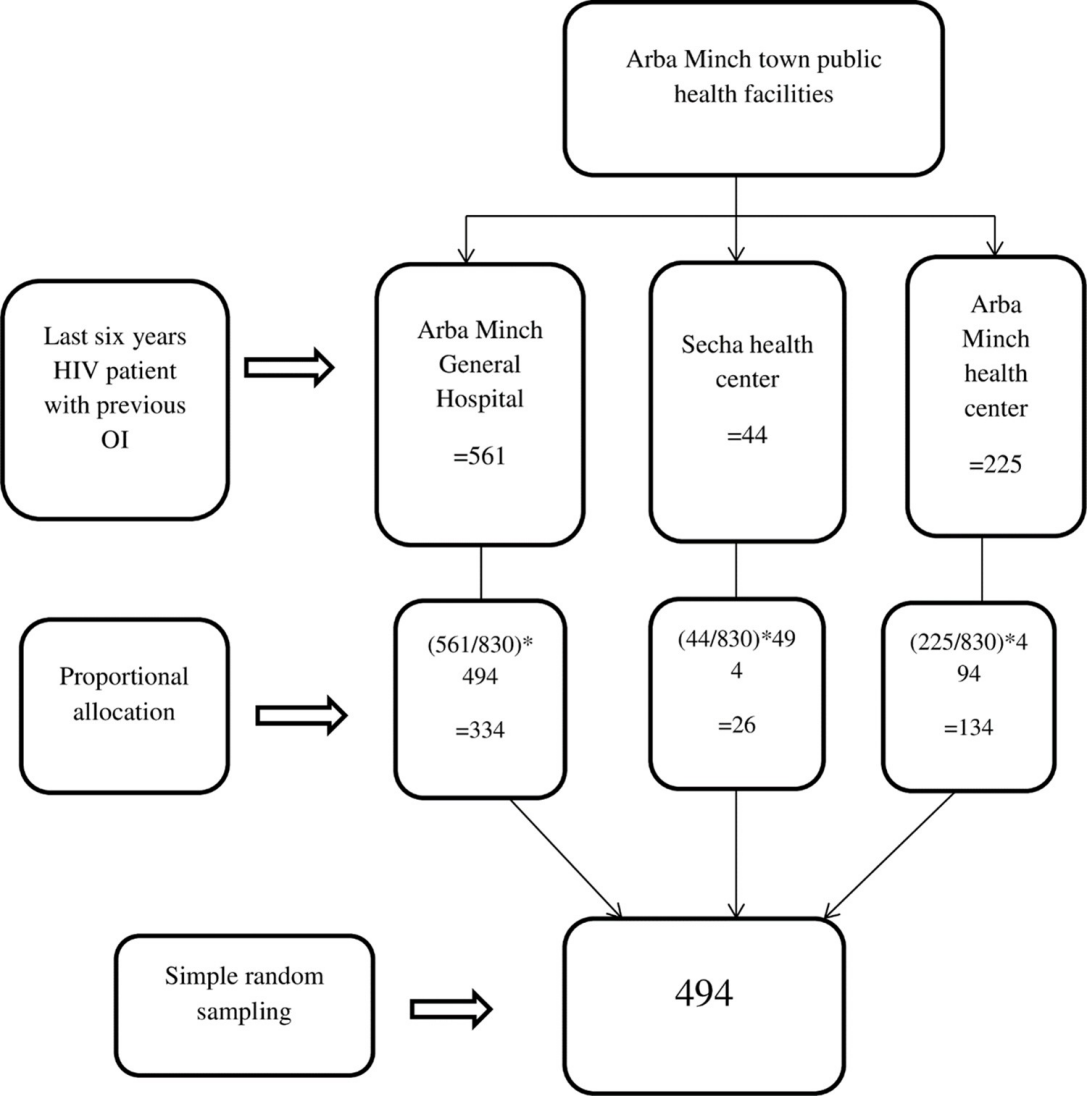
Sampling procedure for current study.

This study includes HIV/AIDS patients who were enrolled into ART clinic from September 2013 up to September 2018. There were 830 HIV/AIDS patients who had previously diagnosed and completed treatment for any of WHO categorized OIs. Sampling frame was prepared for record of these 830 HIV/AIDS patients. For selecting study units, numerical codes were given, and computer generated simple random sampling method was applied. The final sample size was drawn by proportionally allocating the sample based on client flow at respective health facilities (Fig 1). The actual data collection was conducted on 450 (91.1%) complete record of HIV/AIDS patients on ART. All adult 18 and above years of age and who were previously diagnosed and completed treatment for OI were included in the study. On the other hand, transfer in patients and patients on ART treatment for OI but not returned at least once within six months to the health institution for follow up were excluded from the study.

### Data collection procedure and instrument

Data collection checklist was prepared by looking Ethiopian federal ministry of health (FMOH) guideline and individual patient card. The checklist consists of socio-demographic, behavioral, functional status, and clinical and laboratory related variables. Individuals who had opportunistic infection within 6 months of ART initiation were recruited, and the follow up time started from the period of treatment completion of the preceding OI. Thus, HIV/AIDS patients who started ART from September 2013 and who had baseline opportunistic infections will be recruited into the study, and the recruitment will end on September 2018. Recent measurement of variables was considered 6 months before either of reoccurrence, end of follow up, loss to follow up, death, and transfer out was happened. Reoccurrence was measured by reviewing individual patient card for OIs. Those who were on treatment, loss to follow up, died and transfer out during the follow up were considered censored. Based on information from patients’ medical record, clinical and laboratory screening were made to identify possible opportunistic infections; for instance, screening of HIV/AIDS patients for PTB was made by using clinical examination and GeneXpert. For diseases like cryptococcal meningitis, screening was made with lumbar puncture and cerebro-spinal fluid (CSF) analysis. Both the minimum and the maximum follow-up time after completing treatment of initial OI were one year and six and half year.

We discussed the data collection process with public health facilities’ management and ART clinics’ coordinator. Data collectors were 6 BSc nurses who have experience and training on ART service. Three-day training was given for data collectors. Patients’ card were collected from card room, and sampling frame was prepared for those who had previous OIs. Data was collected from 8 April, 2020 to 20 April, 2020.

### Operational definition

**Reoccurrence:** diagnosing of one or more opportunistic infection after treating previous OI.

**Censored:** patient who were loss to follow up, transfer out, complete the study, and died were considered censored.

**Time:** time until reoccurrence of opportunistic infection.

**Good Adherence**: if PLHIV adherent > 95% that is the percentage of missed dose is < 2 doses of 30 doses or <3 dose of 60 dose) as documented by ART health personnel.

**Fair Adherence**: if PLHIV adherent 85–94% that is the percentage of missed dose are 3–5 doses of 30 doses or 3–9 dose of 60 dose) as documented by ART health personnel.

**Poor Adherence**: if PLHIV adherent <85% that is the percentage of missed dose is > 6 doses of 30 doses or >9 dose of 60 dose) as documented by ART health personnel.

### Data analysis and quality control

The data was coded and double entered into Epi data version 3.1, and then it was exported to STATA version 14 (STATA company, Stata Corp, USA, Texas). Before analysis, some continuous variables were changed to categorical variables based on previous studies. Kaplan-Meier survival estimation was done to show time until free of reoccurrence of OIs. Life table was also constructed for the entire cohort of 450 HIV/AIDS patients. Comparison between groups was made by Kaplan-Meier survival curve and log rank test. Assumption of observational independence with cut off value of 10 for variance inflation factor (VIF) was done. Variables like baseline and recent hemoglobin, baseline and recent body mass index (BMI), and baseline and recent CD4 count were checked; there was no multi-collinearity between predictor variables. Finally, assumption of proportional hazard was checked by goodness-of-fit statistical test. Non-significant chi square was considered for not violating the proportional hazard assumption. The statistical significance and strength of the association between independent variable and an outcome variable was measured by bivariate Cox regression model. From this, a variable P value less than 0.2 was transferred to multivariable Cox regression model to adjust confounders’ effects. Model was built and compared by stepwise backwards elimination procedure and likelihood ratio test. A p value less than 0.05 was considered as significantly associated in the final model. Cox-Snell residual was compared against cumulative hazard function to check whether or not the model was fit.

To maintain data quality, training was given for data collectors on objectives of the study, data collection procedure, and confidentiality of the data collection process. Advisors and principal investigator were supervising the data collectors. Incompletely recorded follow up formats were excluded from abstraction. Five percent of the sample was randomly selected and the data were re-abstracted by the supervisor to check the reliability and consistency of data and accordingly correction was made. In addition, at the end of data entry, data cleaning was done using frequencies, cross tabulations, sorting, and listing to check missed values and outliers. Errors identified were corrected by revising the original abstracted format.

### Ethical consideration

A letter of clearance was obtained from institutional research ethics review board (IRB) of Arba Minch University, College of Medicine and Health Science, school of public health. We had been given verbal approval from Arba Minch General Hospital and health centers administration and ART coordinator after explaining the purpose of the study. We also assured that the confidentiality of record review will be kept and no exposition of data at individual level.

The research supervisors and the hospital and health centers ART clinics staffs witnessed the consent process. As this is retrospective document review, we did not receive informed consent from patients. In order to keep the confidentiality of information obtained, we only use medical record number of individual patients to retrieved data from the health institutions data base; we did not use name and anything that expose patient identity. Furthermore, data collectors were ART clinic staffs. So, all these measures helped us to fully anonymize the data before we accessed it.

## Result

### Socio demographic

During the follow up period from Sep 11, 2013 to Mar 9, 2020, a total of 450 (91.1%) HIV patients with previous OI were followed for a median time of 19 months (Interquartile range (IQR) = 7–38). Their mean/ standard deviation (SD) age was 34.3 (±8.47). More than half (58.4%) of the study participant were females. Among the total study participant, 360 (80%) were urban resident. Two hundred fifty-nine (57.6%) were married. One hundred seventy-nine (39.8%) study participants attended primary education, followed by 114 (25. 3%) patients who don’t have formal education. Ninety-seven (21.6%) were government employee by occupation.

### Clinical, laboratory and treatment information’s

In the study, all 450 (100%) patients who diagnosed with one or more opportunistic infections within 6 months were included. Eighty (17.8%) of HIV infected patients were diagnosed with chronic disease. Four hundred twenty-nine (95.3%) of the participant were diagnosed with one opportunistic infection at a time. The study participants’ baseline and recent BMI mean (SD) values were 20.9 (±3.32) and 23.8 (±18.5) respectively. Both baseline and end line median (IQR) CD4 count were 322.5 (183.7–427) and 441.5 (210–630) respectively. Median and IQR values for both baseline and recent hemoglobin were 12.3 (10–13.9) and 12.9 (11–14) respectively.

Majority of them were taking first line regimen in both baseline and at recent visit. Among first line regimen, Tenofovir, Lamivudine and Efavirenz (TDF/3TC/EFV) were the major prescribed drug in base line and on follow up, 416 (92.4%) and 317 (70.4%) patients took these fixed combination drugs, respectively, followed by Tenofovir, Lamivudine, and Nevirapine (TDF/3TC/NVP) during base line and Tenofovir, Lamivudine and delavirdine (TDF/3TC/DTG) during follow up, which account 21 (4.7%) and 73 (16.2%) of patients took these fixed combination drugs, respectively. From OI prophylaxis given, isoniazid was the major one; 37.1% of patients took isoniazid OI prophylaxis. Two hundred fifteen (47.8%) patients were test and treat category. At baseline, 244 (54.2%) were WHO stage 3, 143 (31.8%) were WHO stage 2, and the remaining 63 (14%) were WHO stage 4 HIV patient. One hundred seven (23.8%) had Pulmonary TB, 78 (17.3%) had herpes zoster and 46 (10.2%) were infected with pneumonia and were the major occurred disease at base line. Opportunistic infection reoccurred among 119 (26.4%) HIV/AIDS patients. Based on data from (Table 1) around 92 (77%) cases were reoccurred within 24 months period.

**Table 1:** Life table of cohort of HIV/AIDS patients attending follow-up at public health facilities in Arba Minch Town, Ethiopia.

Interval	Total at beginning	Reoccurred	Censored	Cumulative survival	Hazard rate
0–6	450	31	51	.93	.01
6–12	368	28	51	.85	.01
12–18	289	19	32	.79	.01
18–24	238	14	38	.74	.01
24–30	186	7	26	.71	.01
30–36	153	5	25	.69	.01
36–42	123	8	28	.64	.01
42–48	87	3	26	.61	.01
48–54	58	3	35	.56	.01
54–60	20	1	17	.52	.02
60–66	2	0	2	.52	.00

**Fig 2:**
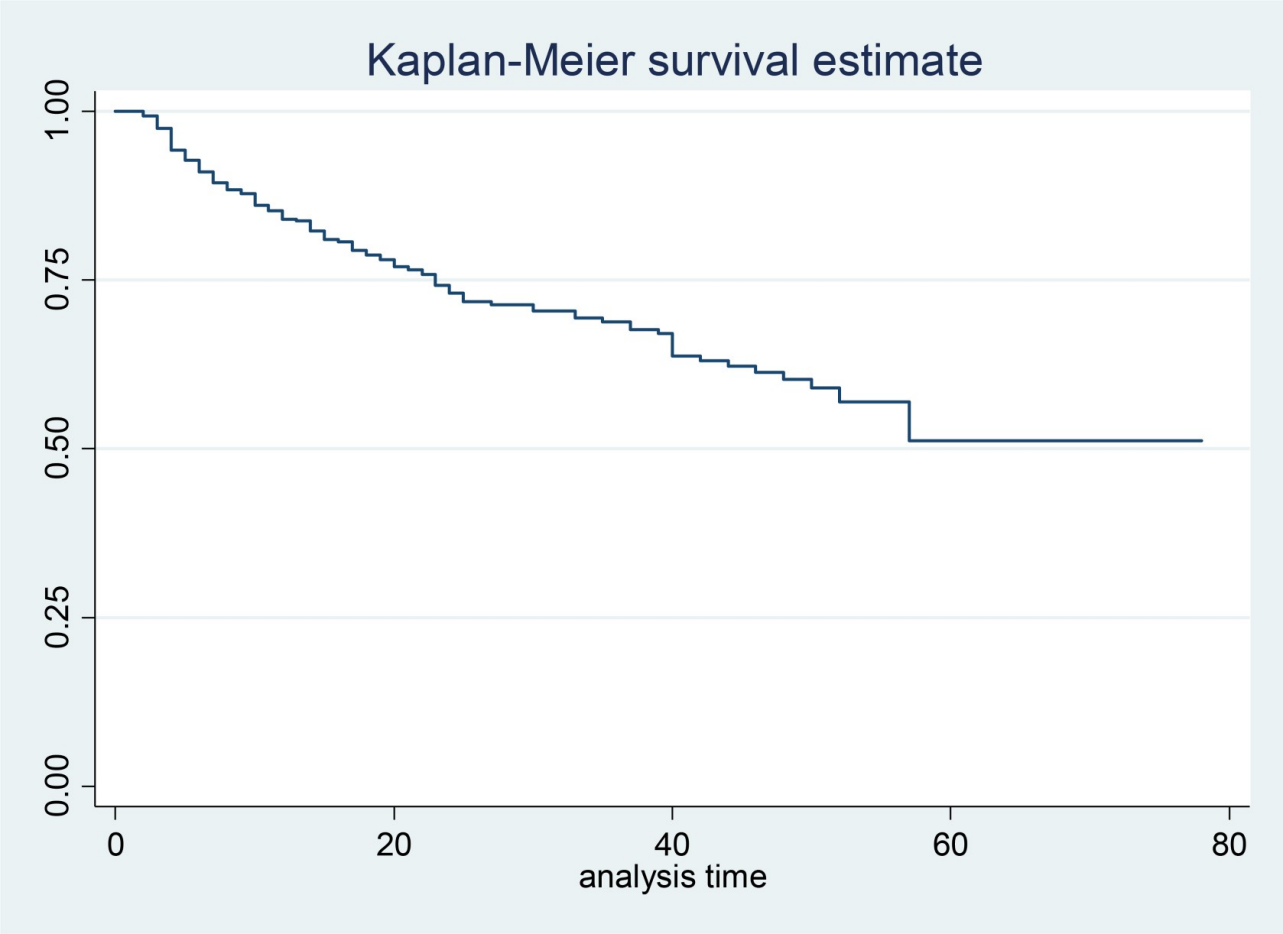
Kaplan-Meier survival estimate for current study.

Kaplan-Meier survival estimate was also conducted for cohort of 450 study participant (Fig 2). The graph shows the cumulative probability of surviving against time.

### Survival analysis

The mean survival time of cohort of 450 HIV/AIDS patients was 52.8 person months. The minimum, the maximum, and the total months taken for the reoccurrence of OI were 1, 78 and 10329 person months respectively. One hundred nineteen patients had reoccurrence of opportunistic infection, with incidence rate of 11.5 (95% CI: 9.6–13) per 1000 person months. The cumulative probability of surviving until 60 months was 52%. Based on data from the life table, around 92 (77%) cases were reoccurred within 24 months’ period. Table 2 shows opportunistic infection that reoccurred among cohort of HIV/AIDS patient. Most frequently occurred OI was pulmonary TB with incidence rate of 6.5 per 100 person months, and the least occurred OI was extra pulmonary TB with the rate of 3.5 per 100 person months.

**Table 2:** Types of OI reoccurred among cohort of HIV/AIDS patient attending ART at public health facility in Arba Minch Town, Ethiopia.

Type of OI reoccurred	Person month of follow up	Failure	IR (%)	95% CI for IR (%)
**Herpes Zoster**	176	6	3.4	(1.53–7.58)
**Diarrheal disease**	107	6	5.6	(2.51–12.4)
**Pulmonary TB**	338	22	6.5	(4.28–9.88)
**Extra pulmonary TB**	85	3	3.5	(1.13–10.9)
**Bacterial pneumonia**	210	12	5.7	(3.24–10)
**Wasting syndrome**	244	17	6.9	(4.33–11.2)
**Oral thrush**	175	13	7.4	(4.31–12.7)
**Chronic diarrhea**	127	8	6.2	(3.15–12.5)
**Genital ulcer**	94	5	5.3	(2.21–127)
**Encephalopathy**	70	5	7.1	(2.97–17.1)
**Toxoplasmosis**	39	7	17	(8.55–37.6)
**Cryptococcus meningitis**	231	15	6.4	(3.91–10.7)
**Total**	1896	119	6.2	(5.24–7.51)

OI, opportunistic infection; IR, incidence rate; CI, confidence interval.

Those variables that were significantly associated with reoccurrence at P- value of ≤ .2 in bivariate analyses were transferred to multivariable analysis. Variables that were associated include base line chronic disease, number of opportunistic infections at base line, base line and recent functional status, base line and recent WHO clinical stage, base line and recent hemoglobin level, and base line and recent CD4 count. In multivariable Cox regression, backward model building method was used until we got statistically significant predators at p-value of ≤.05. After adjusting for confounding effect of covariates, the following variables were significantly associated with the reoccurrence of opportunistic infections. One of the factors which improve the survival probability was body mass index (BMI). Those patients who had BMI of ≥18.5 were 0.46 times less risky (p = 0.039) at any instantaneous point of time as compared to patients with BMI of <16. Those HIV/AIDS patients with CD4 count of >350 were 0.77 times less risky (p = 0.000) at any given time than those patient with CD4 count of <200. Both ambulatory and bedridden functional status patients were 2.3 and 3.4 times high risk (p = 0.008 and p = 0.000) respectively at any given point of time as compared to working functional status. Duration of ART was another significant predictor, in which those who took ART up to three years and more than three years were 0.75 and 0.91 times less risky (p = 0.000 and p = 0.000) respectively at any given point of time as compared to those who took less than one year. Table 3 shows that.

**Table 3:** Multivariable analysis of cohort of HIV/AIDS patients attending follow up at public health facilities in Arba Minch Town, Ethiopia.

Covariates		Reoccurrence of OI		Total (%)	CHR (95%CI)	AHR (95%CI)	P-value
		Yes	No				
Recent Body mass index	<16	24 (20.2)	2 (0.6)	26 (5.8)	1	1	
	16–18.49	29 (24.4)	14 (4.2)	43 (9.6)	.694 (0.403–1.193)	1.55 (0.832–2.892)	.167
	> = 18.5	66 (55.5)	315 (95.2)	381 (84.7)	.093 (0.058–0.150)	0.540 (0.300–0.970)	.039*
Recent CD4+ count	<200	73 (61.3)	35 (10.6)	108 (23.3)	1	1	
	200–350	20 (16.8)	52 (15.7)	72 (15.3)	.355 (0.216–0.584)	0.746 (0.423–1.318)	.313
	> = 351	26 (21.8)	244 (73.7)	270 (60)	.092 (0.062–0.150)	0.226 (0.129–0.397)	.000*
Recent functional status	Working	58 (48.7)	319 (96.4)	377 (83.8)	1	1	
	Ambulatory	31 (26.1)	11 (3.3)	42 (9.3)	7.976 (5.117–12.434)	2.317 (1.249–4.299)	.008*
	Bed ridden	30 (25.2)	1 (0.3)	31 (6.9)	18.556 (11.600–29.684)	3.457 (1.839–6.499)	.000*
Recent hemoglobin	<10	50 (42)	27 (8.2)	77 (17.1)	1	1	
	> = 10	69 (58)	304 (91.8)	373 (82.9)	.205 (0.142–0.297)	.678 (0.429–1.07)	.095
Duration on ART	<1 year	28 (23.5)	66 (19.9)	94 (20.9)	1	1	
	1–3 year	45 (37.8)	136 (41.1)	181 (40.2)	.185 (.108-.315)	.253 (0.141–0.453)	.000*
	>3 year	46 (38.7)	128 (39)	175 (38.9)	.073 (.041-.130)	.093 (0.048–0.183)	.000*

CHR, crude hazard ratio; CI, confidence interval; AHR, adjusted hazard ratio.

*p-value less than 0.05.

## Discussion

In this study, from a total of 494 HIV/AIDS patient records, we analyzed 450 (91.1%) complete records. HIV patients with previous OI were followed for a median time of 19 months (IQR = 7–38). This retrospective cohort study found that 119 patients had reoccurrence of opportunistic infections, with incidence rate of 11.5 (95% CI: 9.6–13) per 1000 person months. OIs reoccurred among 119 (26.4%) HIV/AIDS patients on ART. This finding showed that lower proportion of PLHIV had OIs as compared to previous study conducted on similar topic [11]. This might be due to earlier initiation of ART favors decreased incidence OI. Since in the previous study, study participants were those who are on ART and pre-ART HIV/AIDS patients.

On this study, recent CD4 level was significantly associated with reoccurrence of OI. This finding is in line with other several literatures [11–16]. All studies emphasized that the more the CD4+ count, the less risk of acquiring OI. Patients’ recent BMI was also associated significantly. The study entitled predictive effects of body mass index on immune reconstruction strengthen this concept [17]. Higher BMI could predict better immune reconstruction in HIV patients after highly active anti-retroviral therapy (HAART) initiation. Recent functional status was the other predictor that was associated in this study. This finding was in harmony with the study by *Hunde G* [15]. On another study, which studied the effect of HAART on the incidence of OI, it also pointed out the relation between being bed ridden and OIs development. Bed ridden patients were 4 time higher risk for OI [18]. Based on this, this study comes up as evidence for previous studies in strengthening of their finding. This study also tried to uncover the relation between duration of ART as a predictor factor. This finding is consistent with the other study done in Ghana [19]. This might happen as the duration of ART taken increase the person chance of getting the disease will decrease. Farther more, earlier initiation of ART coupled with longer duration on ART might help the patients cope against being infected with OIs.

But, as a clinical factor, both base line and recent WHO clinical stage were not significantly associated with reoccurrence of OI as with most of the reviewed literature have showed significant association [13, 18, 20]. This might be due to the fact that all the study participants in this study were taking ART at baseline and during follow up time that helped them reduce their chance of acquiring OIs. This might also be due to the fact that earlier studies were affected by treatment initiation delay. Additionally, taking OI prophylaxis played an important role in decreasing the incidence of OIs in the study participants.

As a limitation, this study was affected by incompleteness of patient record. In order to solve this problem, we remove incomplete records from abstraction and adjust the sample size to predict the effect that was really existed.

## Conclusion

This study showed that lower proportion of PLHIV had reoccurrence of OIs as compared to the previous institutional based study. But, as this is incidence study, even a small number of cases have an impact to the larger population group. Predictor factors that were associated with the reoccurrence of OIs include; recent functional status, which was positive factor for the reoccurrence. Recent CD4+ count, recent BMI and duration on ART were negative predictors associated significantly and again were also protective against the reoccurrence.

## Abbreviations

HIV: Human Immune Deficiency Virus
PLHIV: People Living with HIV
WHO: World Health Organization
AIDS: Acquired Immune Deficiency Syndrome
ART: Anti-retroviral Therapy
OI: Opportunistic Infection
CD4: Cell Differentiation 4
PLHIV: People Living with HIV
VIF: Variance Inflation Factor
BMI: Body Mass Index
